# Phosphorus Recovery: New Approaches to Extending the Life Cycle

**DOI:** 10.1289/ehp.119-a302

**Published:** 2011-07-01

**Authors:** Tim Lougheed

**Affiliations:** Tim Lougheed has worked as a freelance writer in Ottawa, Canada, since 1991. A past president of the Canadian Science Writers’ Association, he covers a broad range of topics in science, technology, medicine, and education.


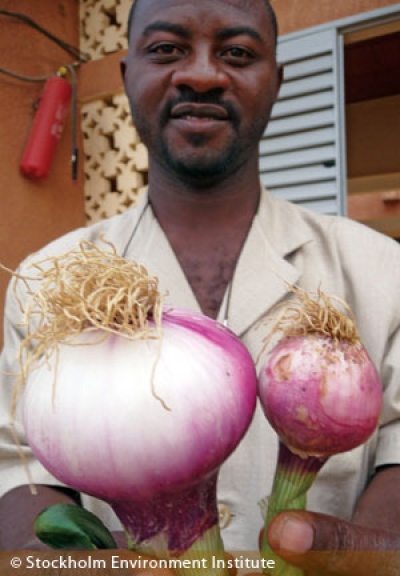
In a May 2011 workshop sponsored by the Department of Energy, phosphorus was considered alongside lithium, neodynium, and rare earth metals as a potentially strategically important element.^1^ Phosphorus, a key element in plant growth (and thus in agriculture), is nonrenewable, obtained for commercial fertilizers from phosphate rock that is mined in a comparatively limited number of sites around the world. Debate swirls around the possibility that viable supplies of this rock will ultimately be exhausted, creating shortages that could devastate global agricultural output and lead to widespread hunger.

Yet the threat posed by phosphorus scarcity contrasts sharply with the current environmental problems created by excessive amounts of the stuff in many places. As a nutrient, this element contributes significantly to algal blooms in waters fed by fertilizer and wastewater runoff. Several new technologies are exploring solutions to limit this impact through recovery or recycling.

## Capitalizing on Struvite

One of those technologies emerged from some complementary consulting work that University of British Columbia civil engineering professor Don Mavinic carried out for a pair of firms in 2000. Metro Vancouver, which operates several wastewater treatment plants in this coastal city, was coping with the buildup of sludge in its pipes. This was not just any sludge, but a concrete-like mass composed of ammonium magnesium phosphate, a mineral that goes by the name struvite. Such deposits, which have been confronted by sewer operators since medieval times, can seriously impede water flow.

Just as Metro Vancouver was asking Mavinic for help, so too did BC Hydro, the power utility that manages dams throughout the mountainous British Columbia interior. The company turned out to have its own interest in struvite, which could replace the commercial product then being used to replenish the nutrient-deficient reservoirs behind those dams. That nutrient compensates for the reservoirs’ reduced populations of fish, whose bodies and wastes provide natural fertilizer to sustain healthy plant growth in lake and river ecosystems.

Mavinic and his colleagues met Metro Vancouver’s request with a reactor that processes wastewater on its way to a biosolids digester. Within a cone-shaped chamber, fine crystals of struvite in the water combine with ammonium, phosphate, and magnesium reagents, growing into particles large enough to capture with a filter. Those particles are the basis for a cost-effective fertilizer that could enable BC Hydro and other dam operators to maintain the environmental integrity of waterways affected by their installations. Government authorities overseeing those waterways, in turn, can ensure that operators sustain the vibrant quality of these settings, which could otherwise turn into biological dead zones.^2^^,^^3^^,^^4^^,^^5^

Recovering and recycling phosphorus in the form of struvite that would otherwise be discarded offers economic as well as environmental incentives. “This high-quality fertilizer struvite is worth its weight in gold,” Mavinic says. “It’s not going to get any cheaper, because as population goes up, you’ve got to feed people.”

The technology developed through the University of British Columbia inspired a spin-off company, Ostara Nutrient Recovery Technologies, Inc. Based in Vancouver, the firm installs facilities to harvest struvite from wastewater streams, marketing their output as a slow-release fertilizer called Crystal Green^®^. The equipment has been installed at wastewater treatment plants in Edmonton, Alberta; Portland, Oregon; and York, Pennsylvania. In 2009, the technology began moving to the other side of the Atlantic, as Ostara inked deals with water authorities in the United Kingdom and the Netherlands.^6^

Companies that run municipal wastewater plants have good reason to consider what Ostara has to offer. Their pipes regularly become clogged with struvite, most of it generated by human urine, which is rich in phosphorus. There is money to be saved in the form of reduced plant maintenance, and money to be made through sales of struvite-based fertilizer. Moreover, in parts of the world where phosphates are in short supply and fertilizers must be imported, this technology could create a domestic source.

Some of the most ambitious struvite recovery targets are being set in Sweden, where the government would like to see 60% of the phosphorus compounds present in the country’s wastewater streams diverted for agricultural use by 2015.^7^ This will not only keep those compounds from draining into lakes and rivers, where they could promote eutrophication, but will also reduce the amount of fertilizer that must be produced or imported.

At the same time, Swedish researchers point out that such strategies need not be limited to the sophisticated wastewater treatment infrastructure of the developed world. The impact could improve human health and economic advantage in many countries around the world. One study considered the United Nations Millennium Development Goals in terms of the cash equivalences of recovering nitrogen and phosphorus from human excreta—in other words, how much money could be saved by recovering phosphorus from local waste streams.^8^ By this analysis, East Asia could retain an annual potential commercial value for these two elements—based on regional costs for fertilizers—totaling more than US$625 million. Similarly, in sub-Saharan Africa this total comes to almost $800 million.

## Better Sanitation Yields Multiple Benefits

Before those savings can be realized, however, it will be necessary to discuss sanitation in practical terms. That happens less often than it should, according to Arno Rosemarin, a senior research fellow with the EcoSanRes Programme of the Stockholm Environment Institute (SEI).

“Although all creatures excrete by-products as part of their natural cycles, humans have managed to turn this into a taboo subject,” Rosemarin says. “As a result, there is a serious lack of political, institutional, and intellectual attention, and more than half the world is grossly suffering for this.”

**Figure:**
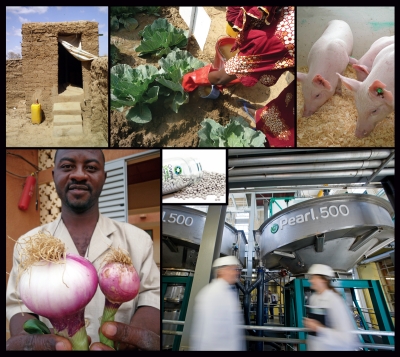
Faces of phosphorus recovery (clockwise from top right): a urine-diverting dry toilet in Niger; using urine to fertilize cabbage; a trio of Enviropigs; Ostara’s nutrient recovery facility in York, Pennsylvania; Crystal Green (inset); onions grown with urine (l) versus chemical fertilizer (r). Clockwise from top right: © Stockholm Environment Institute © Stockholm Environment Institute © University of Guelph © Ostara Nutrient Recovery Technologies, Inc. © Ostara Nutrient Recovery Technologies, Inc. © Stockholm Environment Institute

According to Rosemarin, the general reluctance to discuss sanitation translates into a general neglect of projects in the field, which tend to be poorly designed, poorly installed, and poorly maintained. He says expensive, high-profile attempts to mimic the kind of urban wastewater treatment plants found in European or North American cities are often launched with a great deal of fanfare, but seldom monitored afterward.

A wide variety of pathogens and parasites are found in human excreta, and if this material is ingested, it can result in illnesses ranging from diarrhea to malnutrition, or death.^9^ “Without a dialogue on hygiene and sanitation, the world will remain in a fix,” Rosemarin argues.

For Rosemarin, only part of the problem is represented by the thousands of children who die daily from waterborne diseases that thrive in the absence of basic sanitation.^10^ “What about the fact that 3.5 billion people are infected with helminth worm parasites^9^—a well-kept and dangerous secret?” he asks. “This should be a big deal, at least as big as HIV/AIDS, TB, or malaria. But the global sanitation crisis is not of general knowledge. . . . No political leader has decided to take this one on. The crisis is handled on a piecemeal basis with limited public oversight.”

Peter Morgan, another member of SEI, has explored a number of simple toilet systems that can be built locally and operated far more reliably than large-scale wastewater treatment plants. A major improvement over the primitive pit latrines that are still commonplace in many developing nations, individual outhouse-style toilets follow a life cycle that will see them become composting pits that are eventually planted with trees. Some variations include a diversion for urine, which can be stored and subsequently deployed as a basic fertilizer.

“Urine is a valuable supply of nitrogen and also phosphorus and potassium in smaller quantities,” Morgan says. “It is particularly useful when used to enhance the growth of green vegetables, onions, and maize. It can also considerably enhance the growth of fruit trees like banana and mulberry.”^11^^,^^12^

Above all, urine is free. In places where manufactured fertilizer may be priced far beyond the reach of many farmers—if they can even find it at all—well-managed toilets can provide these same essential elements. As of 2010 the idea had proven persuasive enough to win over the Bill and Melinda Gates Foundation, which gave a $3 million grant to the Swiss Federal Institute of Aquatic Science and Technology (EAWAG) and the eThekwini Water and Sanitation utility in South Africa to study the concept on that continent.^13^

EAWAG has also been honing this same approach in Nepal, where a simplified version of a struvite-extracting reactor has been turning urine into a dry fertilizer that can be applied to fields. Team member Bastian Etter spent two years dealing with the technical challenges of this process, which he acknowledges is still being refined. More satisfying, he notes, is the support the work had found.

“In general, we have experienced an enormous interest for struvite precipitation in Nepal,” Etter says. “Local organizations, media, and individuals welcome this ‘new’ approach linking sanitation and fertilizer production.”^14^^,^^15^

## The Livestock Angle

Meanwhile, back in British Columbia, Mavinic and his colleagues have been coping with an important variation on the toilet taboo—a need to confront the sizeable volumes of manure produced by industrial livestock operations, which can overwhelm ground or surface water with phosphorus.^16^ The Ostara technology might potentially tackle this problem as effectively as it can in municipal wastewater settings, but the smaller scale of most farms could make that a prohibitively expensive proposition. As in Nepal, therefore, researchers are studying a simplified installation that could be more affordable.

Such a solution could also benefit industrial-scale livestock handlers in the United States, who have seen ever-tighter environmental regulations governing concentrated animal feeding operations (CAFOs).^16^ CAFO operators are required to develop nutrient management plans to control the amount of nitrogen and phosphorus discharged from their facilities.^17^^,^^18^ Struvite extraction systems offer one option for nutrient management, with the cost of investment potentially offset by ongoing fertilizer sales. Some observers have suggested that livestock waste could also contribute to growing the feed that sustains these same animals.^19^

Even more daring proposals look at adapting the animals themselves. Since the mid-1990s, researchers at the University of Guelph have been developing a line of genetically modified pigs that synthesize the enzyme phytase in their salivary glands. Phytase helps degrade the phytate that binds phosphorus in plant tissue, enabling the Enviropig™ to utilize far more of the phosphorus contained in the cereal grains and soybean meal it consumes. The pig’s feces consequently contains as much as 75% less phosphorus than that of unaltered pigs on the same diet.^20^

This capability could assign these animals a special status in the highly charged arena of genetic innovation, where public sentiment often borders on suspicion. By 2010 the Enviropig had cleared one of three Canadian regulatory hurdles necessary to allow farmers to raise them by adhering to prescribed production conditions set out by Environment Canada. The remaining steps to be accomplished will be permission for the pigs to be sold as human food and for the nonedible components that normally go for rendering to be used as animal feed.

Cecil Forsberg, one of the initiative’s principal investigators, says this animal’s environmental virtues could overcome public concerns. “Transgenic technology has the potential to enhance the role that the animal industries have in world food production system,” he says. “The unique capability of the Enviropig to efficiently utilize cereal grain phosphorus has the potential to contribute to the sustainability of the global swine industry.”^20^
